# Is Melanoma Progression Affected by Thyroid Diseases?

**DOI:** 10.3390/ijms231710036

**Published:** 2022-09-02

**Authors:** Salvatore Ulisse, Enke Baldini, Daniele Pironi, Federica Gagliardi, Domenico Tripodi, Augusto Lauro, Sabino Carbotta, Danilo Tarroni, Matteo D’Armiento, Aldo Morrone, Flavio Forte, Flaminia Frattaroli, Severino Persechino, Teresa Odorisio, Vito D’Andrea, Eleonora Lori, Salvatore Sorrenti

**Affiliations:** 1 Department of Surgical Sciences, “Sapienza” University of Rome, 00161 Rome, Italy; 2 Scientific Direction, IRCCS San Gallicano Dermatological Institute, 00144 Rome, Italy; 3 Urology Department, M.G. Vannini Hospital, 00177 Rome, Italy; 4 Department of Neurosciences, Mental Health and Sensory Organs, “Sapienza” University of Rome, 00161 Rome, Italy; 5 Laboratory of Molecular and Cell Biology, Istituto Dermopatico dell’Immacolata, IDI-IRCCS, 00167 Rome, Italy

**Keywords:** thyroid diseases, cancer, melanoma, papillary thyroid cancer, BRAF mutations, thyroid hormone, estrogen

## Abstract

Clinical and epidemiological evidence indicate a relationship between thyroid diseases and melanoma. In particular, the hypothyroidism condition appears to promote melanoma spread, which suggests a protective role of thyroid hormones against disease progression. In addition, experimental data suggest that, in addition to thyroid hormones, other hormonal players of the hypothalamic–pituitary–thyroid (HPT) axis, namely the thyrotropin releasing hormone and the thyrotropin, are likely to affect melanoma cells behavior. This information warrants further clinical and experimental studies in order to build a precise pattern of action of the HPT hormones on melanoma cells. An improved knowledge of the involved molecular mechanism(s) could lead to a better and possibly personalized clinical management of these patients.

## 1. Introduction

Benign and malignant thyroid diseases (TD) have been associated with the occurrence of extra-thyroidal malignancies (ETM), including melanoma. In particular, breast and hematological cancers were found to display an increased odds ratio (OR) in all TD, while for other ETM, among which melanoma, colorectal, uterus, kidney, and ovary cancers, the OR augmented significantly in specific TD [1,2,3,4,5,6,7,8,9,10,11,12,13,14,15]. These connections have sparked interest about the possibility of detecting potential common inherited and environmental factors accountable for a disease’s etiology and/or progression. From a clinical point of view, an in-depth definition of such associations may promote an increased surveillance of TD patients at higher risk of developing ETM. On the other hand, elucidation of the underlying molecular mechanism(s) could lead to a better and possibly personalized clinical management of these patients. In this narrative literature review, we examine the epidemiological, clinical, and experimental findings connecting TD and melanoma, with the intention of identifying possible hormonal and molecular mechanism(s) underlying such association. To this end, two authors independently searched for articles in the PubMed database by entering the following keywords in the advanced search builder: melanoma; thyroid disease; (melanoma) and (thyroid disease)/(hypothyroidism)/(hyperthyroidism)/(thyroid cancer)/(thyroid hormones)/(TSH)/(TRH); (melanoma) and (therapy) and (side effects)/(adverse effects). Only articles in English published until July 2022 (including E-Pub ahead of print) were considered. Among these, studies performed on small sample sizes and without statistics were excluded from the discussion of epidemiological and clinical data. In the choice of citations, works published in the last 10 years were preferred, but previous works were also included if they contained relevant information.

## 2. Thyroid Diseases

Thyroid diseases include alterations in gland size (goiter) and/or function (hyperthyroidism, hypothyroidism), inflammatory conditions (thyroiditis), as well as abnormal cell proliferation of benign or malignant nature (adenomas and carcinomas). TDs are very common, showing a higher prevalence in the female gender [16]. Hypothyroidism and diffuse goiter, that may progress into nodular goiter, are usually observed in iodine-deficient areas [16]. On the other hand, autoimmune TD triggering either hypothyroidism, due to chronic lymphocytic thyroiditis, or hyperthyroidism, due to Graves’ disease, are the leading types of TD in iodine-sufficient areas [16]. The occurrence of thyroid nodules is also very frequent and estimated to affect nearly half of the entire adult population [16,17,18]. Up to 95% of thyroid nodules are benign lesions, with the remaining harboring a malignant lesion [17,18,19]. Thus, the first aim of their clinical evaluation is to exclude malignancy [18,19,20,21]. Thyroid carcinomas (TCs) account for roughly 1% of all human cancers, with an incidence sensibly increasing over the last decades as a result of the improved ability to diagnose malignancy in small non-palpable thyroid nodules [18,22,23,24,25,26,27,28,29,30]. The majority of TCs are sporadic, while about 3% to 9% of them display a familial presentation. Familial epithelial thyroid cancers (FETCs) are divided into syndromic and non-syndromic [31,32,33]. Syndromic FETC, with known susceptibility genes, include the Carney Complex, the Werner’s syndrome, the phosphatase and tensin homolog gene (PTEN)-hamartoma tumor syndrome (PHTS), and the familial adenomatous polyposis (FAP) [32,33]. The non-syndromic FETCs are characterized by an autosomal dominant pattern of inheritance with variable penetrance [31,32,33]. Histologically, epithelial TCs include the well-differentiated forms (DTC), comprising the papillary (PTC) and follicular TC (FTC), which may progress towards poorly differentiated TC (PDTC) and incurable anaplastic TC (ATC) [20]. The DTC histotypes represent about 90–95% of all TCs, and over the last years, a great improvement in the comprehension of the molecular mechanisms underlying their progression has been made [34,35]. In particular, more than 98% of oncogenic drivers have been identified, based on which a molecular classification of DTC has been proposed in order to improve patients’ clinical management [34,35]. The most frequent genetic alterations in PTC comprise activating mutations of genes encoding for proteins involved in the mitogen-activated protein kinase (MAPK) signaling pathway (i.e., BRAF and RAS genes), or fusions of RET and NTRK1 genes [31,32]. The progression from DTC to the more aggressive PDTC and ATC is triggered by the onset of additional mutations, such as those of the p53 and the telomerase reverse transcriptase (TERT) genes, and by induction in malignant cells of the epithelial–mesenchymal transition [36,37]. The prognosis of DTC patients is generally satisfactory, with a 10-year-survival rate of approximately 90% [34,37,38,39,40,41,42,43]. However, patients affected by PDTC or ATC do not respond to any treatment currently available, and have a dismal prognosis [36]. For these tumors, new therapeutic approaches based on selective tyrosine kinase inhibitors or immunotherapy are currently under clinical evaluation [41,42,43,44,45,46,47,48,49,50,51,52].

## 3. Melanoma

Melanocytes are skin cells responsible for the formation of melanin, which takes place inside specialized organelles called melanosomes. Melanin is then transferred to keratinocytes, where it concentrates to the apical face of the nucleus to protect the genetic material from ultraviolet radiation damage [53]. Melanoma arises from the malignant transformation of melanocytes and represents the most aggressive skin cancer due to its aptitude to metastasize at the initial stages of tumor growth [54,55,56]. Melanoma incidence and mortality have been found to increase in different world areas, in both men and women [54,55,56,57]. Nearly 90% of melanoma cases are considered sporadic while the remainder are identified as hereditary melanomas, which have an earlier onset with multiple malignant lesions [58]. In 2018 the World Health Organization (WHO) classification, based on specific genetic drivers’ mutations, classified melanomas into nine different categories according to the associated cumulative sun damage (CSD), which relates with molecular alterations of the lesions as reported in Table 1 [59,60,61,62,63,64]. These comprise: (i) low-CSD, represented by superficial spreading melanoma and characterized by a high frequency of the BRAFV600E mutation; (ii) high-CSD, including lentigo maligna melanoma and nodular adenoma, characterized by mutually exclusive mutations of the NRAS, NF1 and BRAF genes except the BRAFV600E; (iii) desmoplastic melanoma, bearing inactivating mutations of the NF1 gene, activating mutations of different proteins involved in the MAPK pathway, or mutations of the NFKBIE gene promoter; (iv) Spitz melanoma, characterized by mutations of the HRAS gene, gene fusion of different kinases such as ROS1, NTRK1 and NTRK3, BRAF, MET and RET, homozygous deletion of the CDKN2A gene, or TERT promoter mutations; (v) acral melanoma, displaying amplification of the CCND1, KIT and TERT genes as well as BRAF, NRAS and KIT gene mutations; (vi) mucosal melanoma, marked by several alterations of gene copy number; (vii) melanoma arising in congenital nevus, characterized by NRAS or BRAF mutations; (viii) melanoma arising in blue nevus, branded by mutations of different genes involved in the Gαq signaling pathway, including the GNAQ, GNA11, CYSLTR2 and PLCB4; and (ix) uveal melanoma, showing mutations similar to those observed in the melanoma arising in the blue nevus [56,57,58,59,60,61,62,63,64,65].

Around 90% of tumors are diagnosed at early stages with no evidence of metastasis. These patients have a favorable prognosis with 10 years’ tumor specific survival ranging between 75 and 95% [56,64]. If diagnosis is made when satellite metastases (up to 2 cm from the primary tumor) and in-transit metastases (between the primary tumor and the first draining lymph node) are present, the 10 years’ survival is reduced to 30–50% [64]. Similarly, the 10 years’ survival of patients with regional lymph node metastases ranges between 40 and 60%. However, patients with distant metastases have a poor prognosis with a median survival of few months [64]. Surgical excision of the lesion represents the primary treatment of melanoma patients followed, when indicated, by radiotherapy of the primary lesion or distant metastases [66]. Adjuvant immunotherapy or targeted therapy may be offered to patients: (i) lacking macroscopic metastases, but at risk of having microscopic metastases; (ii) with lesions thicker than 1.5 mm; or (iii) with completely resected stage II–IV melanoma [66]. In fact, outcomes of clinical trials performed over the last few years demonstrated that adjuvant therapies with ipilimumab, nivolumab, pembrolizumab, dabrafenib, and trametinib in patients with BRAF-mutated melanoma positively affect the overall survival (OS) and/or the relapse-free survival of patients [67,68,69,70,71,72].

## 4. Epidemiological Evidence for Thyroid Disease–Melanoma Association

### 4.1. Benign Thyroid Diseases and Melanoma

Epidemiological works evidenced a positive correlation between the hypothyroidism condition and melanoma progression [14,73,74]. In 2001, Ellerhorst and colleagues, analyzing a case study of 91 patients affected by uveal melanoma, reported a higher prevalence of hypothyroidism (13.2%) of unknown origin in melanoma patients compared to the general population (2%), with no significant effects on stage IV patients’ survival [73]. Later on, the same authors confirmed this finding in a larger case study comprising 1580 cutaneous melanoma patients, for whom the prevalence of hypothyroidism was 7%, and higher in female patients (13.9%) than in male ones (2.4%) [74]. These observations were further corroborated by Shah and colleagues on 156 cutaneous melanoma patients [14]. On the other hand, a population-based cohort study in Sweden performed on 18,156 Graves’ disease patients indicated a decreased risk of melanoma [11]. More recently, in a case study of 6386 female patients affected by benign TD, an augmented risk of ETM, including melanoma, was observed [1]. In particular, an increase in the OR, ranging from 4.8 to 9.1, was recorded for the development of melanoma among patients with benign TD. In addition, the OR was higher in younger patients than in older ones [1]. All together, these observations have led to suppose that alterations of the hypothalamic–pituitary–thyroid (HPT) axis, such as reduced thyroid hormones (TH), increased thyroid stimulating hormone (TSH), or thyrotropin releasing hormone (TRH) levels, could promote melanoma growth. The rationale and the evidence supporting this hypothesis will be described below.

In addition to arising spontaneously, TD can be induced by therapeutic regimens with several non-thyroid related drugs. Among these, there are immune checkpoint inhibitors (ICI), i.e., monoclonal humanized antibodies that interfere with the immune self-tolerance, resulting in a stimulation of T-cells to destroy the cancer cells [74,75]. Treatment with ICI greatly improved OS rates in unresectable and completely resected advanced-stage melanomas, as well as in adjuvant settings [76,77]. At present, antibodies targeting the cell surface programmed cell death 1 (PD-1) receptor (nivolumab or pembrolizumab), either in monotherapy or in combination with an antibody against the cytotoxic T-lymphocyte antigen-4 (CTLA-4) (ipilimumab), are the standard of care for melanoma patients [78]. In 2015, a systematic review and meta-analysis was performed on a total of 1265 patients from 22 clinical trials employing anti-CTLA-4 antibodies to assess the incidence of immunotherapy related adverse events (irAE) [79]. Autoimmune hypophysitis was the most common endocrine side effect, reported in up to 13% of clinical trials, with a main impairment of the HPT axis (89.3%). Conversely, hypo- and hyperthyroidism secondary to thyroiditis were infrequent (up to 5.6% in clinical trials). At the same time, another study evaluated survival as primary end point, and a series of irAE as secondary end points, on 945 patients with unresectable stage III or IV melanomas which received nivolumab or ipilimumab or both [80]. Hypothyroidism, hyperthyroidism, and hypophysitis were observed in a few patients, from 15% to less than 1% depending on the drug and the TD, but in almost all cases these effects were mild or moderate. More recently, another systematic review and meta-analysis estimated irAE in 6331 patients with advanced melanoma treated with anti-CTLA-4 or anti-PD-1 antibodies as monotherapy or in any concomitant or sequential combination [81]. The results obtained evidenced that hyperthyroidism, hypothyroidism, hypophysitis, and thyroiditis were the most frequent endocrine irAE. A very recent work examined the influence of thyroid dysfunctions consequent to anti-PD-1 therapy on OS rates of 249 melanoma patients [78]. Interestingly, clinically significant hypothyroidism was found to be an independent prognostic factor, with Hazard Ratio (HR) 0.51 (95% CI 0.29–0.87). This observation conflicts with the findings obtained for spontaneous onset hypothyroidism, which appears to be positively associated with melanoma progression, as mentioned above. However, in the case of drug-induced hypothyroidism, the better prognosis of melanomas may not depend on alterations of the HPT axis, but rather on the greater efficacy of the immunotherapy itself, which is also manifested in the form of irAE impacting the thyroid. In line with this hypothesis, one study executed on patients treated with single-agent anti-PD-1 antibodies for advanced cancers of various origins documented a markedly improved response to therapy in patients with irAE over those without irAE [82]. Moreover, in patients receiving tyrosine kinase inhibitors for different malignancies, including melanoma, new-onset hypothyroidism was associated with favorable clinical response [83].

### 4.2. Malignant Thyroid Diseases and Melanoma

Several studies documented a clear association between thyroid cancers and melanoma [1,7,15,84,85,86,87,88,89,90]. Patients with melanoma have a 2.3-fold higher risk of developing a PTC, while patients with PTC have a 1.8-fold higher risk of getting melanoma [84]. The reasons of such association remain to be determined. The fact that both melanoma and PTC are frequently characterized by activating mutations of the BRAF gene suggests the presence of shared etiologic events [15,91,92,93]. In both tumor types, the BRAF mutations, particularly BRAF^V600E^, are often encountered in more aggressive phenotypes [15,90,91,92]. In addition, germline mutations capable of conferring susceptibility to both melanoma and thyroid cancer have been identified [89,90,91,92]. Among these are the inactivating mutations of the PTEN gene responsible for the Cowden syndrome, characterized by multiple hamartomas and increased risk of breast, thyroid, endometrial, colorectal, and renal cancers, as well as of melanoma [94]. Germline mutations in the protection of telomeres 1 (POT1) gene have been shown to be implicated in melanoma predisposition [95,96,97,98,99,100]. Recently, a germline POT1 mutation was also identified in a two-generation Italian family with familial non-medullary thyroid cancer (FNMTC) [98]. However, a subsequent study performed on patients from seven families with non-syndromic FNMTC failed to detect any mutations in the POT1 gene [99]. Germline mutations in the BRCA-1 associated protein (BAP1) tumor suppressor gene underlie a tumor susceptibility syndrome characterized by increased risk uveal melanoma and melanocytic tumors (i.e., benign and malignant melanocyte-derived tumors) [100]. Family members carrying the germline BAP1 mutation c.1777C>T, which produces a truncated BAP1 protein product, showed early-onset melanocytic neoplasms and other neoplasia, including thyroid cancer [100].

## 5. Hormonal Players of the Hypothalamic–Pituitary–Thyroid Axis and Melanoma

As described below, different hormonal players of the HPT axis are thought to be involved in melanoma progression (i.e., TRH, TSH, and TH).

### 5.1. Thyrotropin-Releasing Hormone and Melanoma

**Figure 1:**
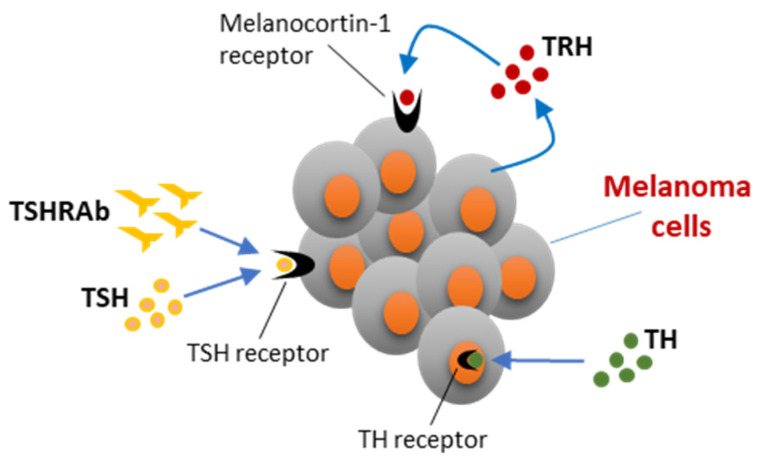
Schematic representation of the action of the different hormonal players of the hypothalamic–pituitary–thyroid axis on melanoma cells. TRH, thyrotropin-releasing hormone; TSH, thyroid stimulating hormone; TSHRAb, TSHR autoantibodies; TH, thyroid hormones. See description in the text.

Although the pituitary thyrotropin-releasing hormone (TRH) does not enter the systemic circulation, the presence of immunoreactive TRH has been found in melanoma tissues, with its expression thought to be induced by low levels of TH as occurs in the hypothalamus [101,102,103]. Furthermore, it was shown that the expression of TRH was more likely to be present in dysplastic nevi than in normal nevi, and that dysplastic nevi from melanoma patients had a higher TRH expression compared to those of healthy subjects [103]. As illustrated in Figure 1, the hypothesis that locally produced TRH could induce melanoma growth relies on its ability to bind and activate the melanocortin-1 receptor expressed in both melanocytes and melanoma cells [103,104,105]. Indeed, Ellerhorst and colleagues demonstrated a stimulatory effect of low TRH concentrations on melanoma cells proliferation [103]. In this context, it is also worth mentioning the observations reported by Gáspár and colleagues in organ-cultured human hair follicles (HFs) expressing both TRH and its receptor [105]. They showed that TRH stimulated melanin synthesis, transcription and activity of tyrosinase (an enzyme implicated in the synthesis of melanin), melanosome formation, proliferation, and dendricity of isolated HF melanocytes. Altogether, these findings support the potential regulatory role of TRH in melanocyte function and melanoma progression.

### 5.2. Thyroid-Stimulating Hormone and Melanoma

In view of the increased prevalence of hypothyroidism among melanoma patients, the possibility that elevated levels of thyroid-stimulating hormone (TSH) could promote the growth of melanoma cells was also taken into consideration. Indeed, TSH has been recognized as a tumor promoting factor not only for DTC, but even for other ETM such as ovarian cancer and hepatocellular carcinoma [106]. TSH receptor (TSHR) expression, at both mRNA and protein level, has been documented in skin tissues and different skin cell types including cultured keratinocytes, dermal fibroblasts, epidermal melanocytes, and melanoma cells [102,107,108,109,110]. In particular, Ellerhorst and colleagues detected the TSHR in all cutaneous melanocytic lesions, namely benign nevi, dysplastic nevi, and melanomas [109]. This receptor was proven to be functional, as demonstrated by the ability of TSH to stimulate, in melanoma cells, cAMP formation and the MAPK pathway. In addition, cultured melanoma cells, but not melanocytes, were prompted to proliferate by physiological TSH concentrations (see Figure 1) [109,110]. These results clearly indicate a role for TSH in melanoma progression. However, the above-mentioned Sweden population-based cohort study recorded a decreased risk of melanoma in patients affected by Graves’ disease [11]. A possible explanation for this discrepancy could be that activating TSHR autoantibodies, characterizing Grave’s disease, fail to activate the TSHR expressed on human melanocytes; otherwise, TH excess produced in Grave’s disease patients could have a dominant protective role against melanoma progression, as explained below (see Figure 1).

### 5.3. Thyroid Hormones and Melanoma

#### 5.3.1. A Brief Overview of Molecular Mechanisms Underlying Thyroid Hormones Action

Thyroid hormones (TH) represent major supervisors of body growth and development and modulate a number of homeostatic cellular functions in adults [111]. The two TH, 3,5,3′,5′-L-tetraiodothyronine (thyroxine, T_4_) and the 3,5,3′-L-trioiodothyronine (T_3_), act on target cells in two main modes, genomic and non-genomic, which may partly overlap [111,112].

In the classical genomic action, TH enter target cells through specific plasma membrane transporters (i.e., the monocarboxylate transporters MCT8 and MCT10, the organic anion transporters OATP1 and OATP3, and the L-type amino acid transporter LAT) [113]. Inside the cytoplasm, T_4_ is deiodinated by the deiodinases 1 (D1) or 2 (D2) to form T_3_, which binds the TH nuclear receptor (THR) with greater affinity compared to T_4_ [111]. The THR binds to specific DNA sequences within promoter regions of target genes, known as thyroid responsive elements (TRE), increasing or inhibiting transcription [114,115,116]. Two THR proteins are encoded by separate genes: the THRA, located on chromosome 17, and THRB, located on chromosome 3 [117,118,119]. The THRA gene generates three different transcripts, THRα1, THRα2, and THRα3, of which only the THRα1 is translated in a protein able to bind T_3_ [120,121]. THRα2 and THRα3 receptors can heterodimerize with THRα1 and are thought to antagonize T_3_ effects on gene transcription [120,121]. The THRB gene encodes two isoforms, THRβ1 and THRβ2, both of which bind T_3_ but differ in their tissue distribution [116].

The non-genomic actions of TH arise from the interaction with non-nuclear receptors, either structurally related to THR or not, located in mitochondria, cytoplasm, or plasma membrane. In the latter, T_4_ and T_3_ may bind to specific sites of the integrin αvβ3, termed S1 and S2 [112,113,122]. The S1 site binds only T_3_ and stimulates the phosphatidylinositol 3-kinase (PI3K), while the S2 binds mainly T_4_ inducing the extracellular signal-regulated kinases (ERK) 1 and 2. Further non-genomic effects identified so far are the regulation of the cytoskeleton, the stimulation of Na,K-ATPase and Ca^2+^-ATPase activities, and the activation of signal-transducing proteins and nitric oxide synthase (NOS) that ultimately increase cell proliferation [112].

#### 5.3.2. Thyroid Hormones’ Effects on Melanoma Development

At present, information about the effects of TH on normal human melanocyte development is very limited, but the role of TH was explored in zebrafish melanophore development [123,124]. In particular, McMenamin and colleagues reared zebrafish larvae in T_4_, and observed a melanophore deficiency [123]. This phenotype was similar to the mutant *opallus^b1071^*, which was recognized as carrying a missense activating mutation (Asp632 → Tyr) of TSHR. In contrast, the induction of hypothyroidism caused a marked increase in melanophore number in these animals [123]. It is thus clear from this study that TH have antiproliferative and differentiating effects of TH on zebrafish melanophores [123,124]. In line with these experimental observations are those of Di Cicco and colleagues, obtained in patients affected by resistance to TH due to mutations in the THRA gene [125]. These patients are characterized by signs of hypothyroidism and exhibit numerous skin lesions. Histological and molecular analyses of skin nevi from four of them evidenced the expression of keratin 17 and type 3 deiodinase, indicative of unbalanced, increased cellular proliferation versus differentiation, along with a higher expression of molecular markers suggestive of different skin malignancies, including melanoma (Figure 1) [125]. All together, these data appear to fit well with those of Ellerhorst and colleagues, reported above, showing an increased prevalence of hypothyroidism among melanoma patients, as well as with the outcome of a Sweden cohort study on patients receiving levothyroxine therapy [126]. These patients, while displaying a slight but statistically significant increase of the overall cancer risk associated with treatment in both men (adjusted HR 1.06, 95% CI 1.03–1.10) and women (adjusted HR 1.08, 95% CI 1.07–1.10), had a reduced HR for melanoma, particularly men (HR 0.78, 99% CI 0.64–0.96) [126].

A number of pieces of experimental evidence indicate that estrogens may influence cutaneous melanoma growth and progression [127,128,129,130]. In particular, a possible protective role of estrogens against melanoma has been hypothesized following the observation of a higher incidence of melanoma in men than in women, combined with a significant survival advantage in women, and of a lower probability of localized melanomas to form metastases in women than in men [131,132,133,134]. This gain in survival is apparently limited to women under the age of 60, when the estrogen levels decline following menopause [135]. Estrogen action is mediated by three different receptors: the estrogen receptors alpha (ERα) and beta (ERβ), and the G-protein coupled estrogen receptor (GPER) [129]. The GPER level is inversely related to patients’ OS and has been associated with melanoma cells differentiation and inhibition of proliferation, as well as with an increased susceptibility to immune-mediated elimination of malignant cells [130,136]. ERα expression was appreciated at the early tumor stage, and decreased with the increasing tumor thickness, while that of ERβ was detected in all melanocytic lesions and remained unchanged during melanoma progression [129,130]. Interestingly, selective inhibition of ERβ negatively impacted the prometastatic behavior of melanoma cell models. As a consequence, ERβ has been identified as a potential therapeutic target for advanced melanoma [129,130,137]. TH could affect estrogen action in several ways. First, TH may enhance the expression level of both ERs, especially ERα [138,139]. Moreover, the TRE and the ER responsive element (ERE) share an identical half-site, and THRs were shown to bind also to ERE [140]. Finally, through the αvβ3 integrin receptor, TH may activate MAPK signaling and phosphorylation of the nuclear ERα [141]. The ER phosphorylation status affects its ability to interact with chromatin, to recruit coregulators, and to modulate gene expression even in the absence of estrogen [141,142,143].

## 6. Conclusions

To the best of our knowledge, epidemiological, clinical, and experimental data so far available in the literature strongly suggest a role of TH in the progression of melanoma. However, further studies are needed to fully characterize TH effects at the molecular level in normal and malignant melanocytes. For example, no information is yet available on the expression and functions of the αvβ3 integrin, a plasma membrane TH receptor shown to play a role in other cancer types, in melanomas. A deeper characterization of the TH actions on melanocytic lesions could pave the way for new therapeutic approaches, and possibly for a renewed and personalized clinical management of melanoma patients.

## Figures and Tables

**Table 1:** Melanoma classification according to the associate cumulative sun damage (CSD) that relates with lesion’s molecular alterations. See explanation in the text. Adapted from reference [59] with permission.

Melanoma Classification	
*Melanoma arising in* *sun-exposed skin.*	1. Low-CSD melanoma/superficial spreading melanoma
	2. High-CSD melanoma/lentigo maligna melanoma
	3. Desmoplastic melanoma
*Melanoma arising at* *sun-shielded sites or without known etiological association with UV radiation exposure.*	4. Malignant Spitz tumor (Spitz melanoma)
	5. Acral melanoma
	6. Mucosal melanoma
	7. Melanoma arising in congenital naevus
	8. Melanoma arising in blue naevus
	9. Uveal melanoma
